# The Efficacy of Quadruple Therapy Versus Triple Therapy in Helicobacter pylori Eradication

**DOI:** 10.7759/cureus.82255

**Published:** 2025-04-14

**Authors:** Abdulrahman Barakat, Rawan Althahabi, Hanin Aljazaf, Mariam Hammadi, Mohamed Alabbasi, Faisal Alserdieh, Omar Sharif, Faisal Abubaker

**Affiliations:** 1 Internal Medicine, King Hamad University Hospital, Muharraq, BHR; 2 Gastroenterology, King Hamad University Hospital, Muharraq, BHR

**Keywords:** drug efficacy, helicobacter pylori eradication, hpylori, quadruple therapy, triple therapy for h. pylori

## Abstract

Background and aims

*Helicobacter pylori* (*H. pylori*) is a significant cause of various gastrointestinal diseases, including gastritis, peptic ulcers, and even stomach cancer. Therefore, eradication is essential to prevent these conditions and their associated complications. The emergence of antibiotic-resistant strains and the lack of sufficient data on antibiotic sensitivity have made it increasingly challenging to treat *H. pylori*, which has led to the emergence of various regimens. This article will compare the efficacy of different regimens to provide insights into the most effective treatment options for *H. pylori*.

Methods

Our study is a retrospective, single-center study that reviews the data of 1,246 outpatients who were diagnosed with *H. pylori* infection and underwent testing to confirm eradication of *H. pylori* after receiving the therapeutic regimen. Patients received one of the following regimens: clarithromycin-based triple therapy (CT), levofloxacin-based triple therapy (LT), or bismuth-based quadruple therapy (BT).

Results

In total, 1,246 individuals were treated with different regimens, resulting in an overall success rate of 69.3%. The success rates of the triple and quadruple therapies were comparable, with 67.7% and 74.3%, respectively. When looking at individual treatment regimens, BT had the highest success rate at 74.3%, followed by CT with 67.9% and LT with 64.8%.

Conclusion

In Bahrain, BT demonstrates greater effectiveness compared to commonly used triple therapies. Despite this, the overall eradication rate remains low, indicating a significant presence of resistance within the population. Therefore, it is crucial to emphasize the need for sensitivity testing to develop local antibiograms and ensure cost-effectiveness.

## Introduction

*Helicobacter pylori* (*H. pylori*) is a common bacterium that can infect the stomach and cause various gastrointestinal diseases, including gastritis, peptic ulcers, and even stomach cancer. Although prevalence has decreased in many regions following the increased use of eradication therapy and improved sanitary conditions, a study published by Li et al. (2023) suggests that more than 40% of the global adult population remains infected with *H. pylori* [1]. Another study by Hooi et al. (2017) demonstrated that *H. pylori* affected 4.4 billion people globally in 2015, with incidence rates varying widely across regions. While Switzerland had a relatively low incidence rate of 19%, Nigeria had a significantly higher incidence rate of 88% [2].

Several factors contribute to the high prevalence of *H. pylori* globally. Poor sanitation, overcrowding, and low socioeconomic status are significant risk factors, especially in developing countries. The infection is more common in areas with inadequate access to clean water and hygiene facilities. Lifestyle factors such as smoking, alcohol consumption, and unhealthy diets also increase the risk of infection. In addition, the overuse of antibiotics and proton pump inhibitors (PPIs) can lead to an increase in antibiotic-resistant strains of *H. pylori*, making it more difficult to treat. Overall, a combination of environmental, lifestyle, and healthcare-related factors contributes to the high prevalence of *H. pylori* worldwide [3].

According to a retrospective study conducted in the Kingdom of Bahrain, the prevalence of *H. pylori* infection among patients with dyspepsia is approximately 22.4% [4]. The study also found that the prevalence of *H. pylori* was higher in men, with 83.1% of males being positive for the infection [5]. This suggests that *H. pylori* is a significant health concern in Bahrain, and early diagnosis is crucial to prevent serious conditions resulting from longstanding infections.

*H. pylori* is considered an infectious disease; therefore, treatment should be provided to all patients, regardless of symptoms [6]. Proper treatment has proven to be crucial in reducing the occurrence of peptic ulcer disease and gastric cancer [7]. Eradicating *H. pylori* was shown to decrease the risk of gastric cancer by nearly two to five times [8-10]. Additionally, a study confirmed that eradicating *H. pylori* reduces mortality from gastric cancer [9].

A rise in the prevalence of *H. pylori* antibiotic resistance has been attributed to the overuse of antibiotics and improper use of medications. Recently, there has been a significant decrease in the efficiency of standard triple therapy by up to seven times in patients due to clarithromycin resistance. Furthermore, from 2006 to 2016, resistance to metronidazole and levofloxacin, two antibiotics used in triple therapy, has surpassed the 15% mark in several World Health Organization (WHO) regions. This is due to the overuse of these antibiotics in treating different infections, with a 64% increase in global fluoroquinolone usage from 2000 to 2010. Hence, using levofloxacin to treat *H. pylori* in these patients was associated with a higher risk of eradication failure by around eight times [11].

In Bahrain, the prevalence of resistance among 83 *H. pylori* isolates that were cultured from biopsies during routine endoscopies between 1998 and 1999 was evaluated through a study, which showed that 57% of the strains (n=47) were resistant to metronidazole, while 32.5% of the strains (n=27) were resistant to clarithromycin [12]. Therefore, it is important to implement unified national treatment policies by studying local trends of antibiotic resistance to reduce rates of eradication failures from the first attempt and, in turn, reduce the prevalence of *H. pylori* and prevent further complications.

The development of alternative regimens, including bismuth-based quadruple therapy (BT), sequential therapy, and concomitant therapy, has played a significant role in treating *H. pylori* infections. Our study aims to evaluate and compare the efficacy of clarithromycin-based triple therapy (CT), levofloxacin-based triple therapy (LT), and BT as the first treatment for *H. pylori *infection.

## Materials and methods

Patient recruitment

This retrospective study reviewed medical records of outpatients who attended the gastroenterology clinic at King Hamad University Hospital, Kingdom of Bahrain, between January 2017 and December 2021. Data were extracted from the hospital's electronic health record (EHR) system, which securely stores clinical and diagnostic information. Data collected included demographics, diagnostic test results, treatment regimens, reported compliance, and outcomes of follow-up eradication tests.

A total of 2,060 patients initially met the inclusion criteria: (1) confirmed *H. pylori* infection via urea breath test or stool antigen test, and (2) received first-line triple or quadruple therapy for *H. pylori*.

Of these, 814 patients were excluded due to a lack of confirmatory follow-up testing, which was required to assess treatment efficacy. These cases were excluded either due to loss to follow-up, patients not returning for post-treatment testing, or incomplete documentation. The final analysis included 1,246 patients.

Diagnosis

Infection with *H. pylori* was confirmed through two noninvasive tests: the urea breath test and the helicobacter stool antigen test. Patients were asked to stop PPIs at least two weeks prior to testing, and it was ascertained that they had not received any antibiotics at least four weeks prior to the tests. Testing for eradication was performed using the same initial test for each patient individually, at least four weeks from the last dose of antibiotics in their therapy regimen.

The HEADWAY© 13C-UBT kits (Headway Bio-Sci & Tech Co., Ltd., Shenzhen, China) were employed for the urea breath test, which has a 95% sensitivity and a 96% specificity. The HP stool antigen cassettes (TECO Diagnostics, Anaheim, CA, USA) used yielded a 95% sensitivity and a 94% specificity [13].

Treatment regimens

We selected the three most prescribed anti-*H. pylori* regimens at the gastroenterology clinics for further investigation: CT, LT, and BT. CT was composed of clarithromycin 500 mg orally twice daily, amoxicillin 1000 mg orally twice daily, and omeprazole for 14 days. LT was composed of levofloxacin 500 mg orally once daily, amoxicillin 1000 mg orally twice daily, and omeprazole for 14 days. BT was composed of bismuth subcitrate potassium 420 mg, metronidazole 375 mg, tetracycline hydrochloride 375 mg in the form of three capsules, four times daily for 10 days (Pylera® was used, Allergan Pharmaceuticals, Dublin, Ireland), and omeprazole for at least 14 days.

The choice of antibiotic regimen was made individually by the physician attending the clinic at the time, influenced mainly by the history of penicillin allergies, history of adverse effects, and the availability of the antibiotics in the facility. Compliance was defined as the use of antibiotics for at least 10 days. Successful eradication was determined by a negative test performed four weeks or more after treatment completion.

Research ethics

The Institutional Review Board of King Hamad University Hospital reviewed and sanctioned this investigation (ref. no. 22-485). Given the retrospective nature of the study, the study subjects were not requested to provide written informed consent.

Statistical analysis

All data were coded and entered into IBM SPSS Statistics for Windows, Version 25 (Released 2017; IBM Corp., Armonk, New York). Descriptive statistics were used to summarize patient demographics and treatment outcomes. Categorical variables were presented as frequencies and percentages, while continuous variables were presented as means ± standard deviations.

Chi-square (χ^2^) tests were used to assess associations between treatment regimens and eradication outcomes, as well as to examine the relationship between eradication success and confounding variables such as age, gender, and compliance. The choice of the chi-square test was appropriate due to the categorical nature of the primary outcome variables (eradication: yes/no; regimen type: CT/LT/BT). A p-value < 0.05 was considered statistically significant.

To ensure reliability and validity, only patients with clearly documented diagnostic and follow-up testing were included. All *H. pylori* tests were performed in the hospital's certified laboratories using validated commercial kits (HEADWAY© 13C-UBT and *H. pylori* stool antigen cassettes), both of which are known to have high sensitivity and specificity. All laboratory procedures adhered to manufacturer protocols and were conducted by trained personnel.

## Results

The baseline characteristics of the included patients, the diagnostic tool, and the treatment regimen used are presented in Table 1.

**Table 1:** Population demographics

Variable	Sub-variable	Frequency	Percentage
Gender	Male	472	37.8%
	Female	774	62.2%
Diagnostic Tool	Urea Breath Test	1103	88.5%
	Stool Antigen	143	12.1%
Treatment Received	Triple Therapy	837	66.2%
	Quadruple Therapy	409	32.3%
Antibiotics	Clarithromycin Based	783	62.9%
	Levofloxacin based	54	4.3%
	Bismuth based	409	32.8%
Course Completed	Yes	1110	89.1%
	No	72	5.8%
	Missing	64	5.1%
Eradication	Negative Result	871	69.9%
	Positive Result	375	30.1%

The mean age of the participants was 44.98±15.45, with a minimum age of seven and a maximum age of 91. The majority of the participants were females (n=789), accounting for 62.2% of the total number of patients. All the patients were treated for the first time, with CT being the most common first-line treatment regimen administered to 62.9% of patients (783/1246), followed by BT (409/1246, 32.8%) and LT (54/1246, 4.3%). Among these, all the patients in BT were treated for 10 days, while in CT and LT, participants received the treatment for 14 days.

During follow-up visits, 89.1% of patients reported compliance with the complete regimen course, while 5.8% (n=72) reported non-compliance with therapy due to missed doses or discontinuation of drugs due to adverse effects. Of the 72 patients who were non-compliant with therapy, only 25 (34.7%) achieved successful eradication, while 47 (65.3%) had persistent infection. In contrast, among compliant patients, the eradication rate was 76.2% (846/1110). This difference was statistically significant (p < 0.0001), indicating that compliance is strongly associated with successful eradication of *H. pylori*.

The overall eradication rate for all treatment regimens was 69.9% (871/1246), while in 30.1% of patients, the treatment failed to eradicate *H. pylori *(375/1246). Treatment efficacy ranged from 64.8% to 74.3%, depending on the antibiotic regimen used. Table 2 presents the comparison of the different regimens used.

**Table 2:** Comparison of different regimens used *p-value < 0.05 was considered statistically significant.

Variable		Bismuth-Based Quadruple Therapy (n=409)	Clarithromycin-Based Triple Therapy (n=783)	Levofloxacin-Based Triple Therapy (n=54)	p-value
Age		Mean: 46.70±15.57 Range: 84 (7-91)	Mean: 43.85±15.18 Range: 76 (8-84)	Mean: 47.35±15.46 Range: 62 (20-82)	0.439
Gender	Male	148, 36.2%	305, 38.9%	19, 35.2%	0.592
	Female	261, 63.8%	478, 61.1%	35, 64.8%	
Course Completed	Yes	351, 85.8%	704, 89.9%	49, 90.7%	0.034*
	No	33, 8.1%	35, 4.5%	4, 7.4%	
	N/A	25, 6.1%	44, 5.6%	1, 1.9%	
Eradication	Negative Result	304, 74.3%	532, 67.9%	35, 64.8%	0.052
	Positive Result	105, 25.7%	251, 32.1%	19, 35.2%	

**Figure 1:**
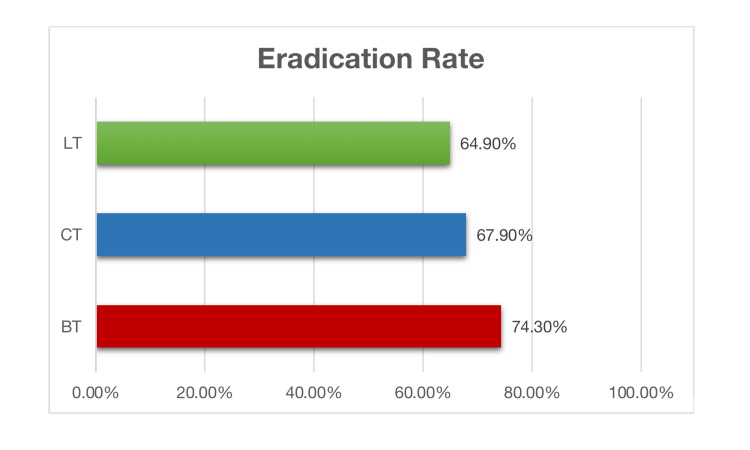
Eradication rate

The completion of treatment was significant in the population (p=0.034), and eradication was marginally significant (p=0.052) (Table 2). The most used CT had an efficacy rate of 67.9% (532/783) for a 14-day course. BT, being the second most common therapy, had a higher eradication rate of 74.3% (304/409). LT was successful in 64.8% of cases for 14-day courses, but the results were not statistically significant due to the small number of patients (Figure 1).

There was no difference (p=0.439) between the average ages of the groups, which were 43.85±15.18, 47.35±15.46, and 46.70±15.57 in the CT, LT, and BT groups, respectively. The male ratios were 38.9%, 35.2%, and 36.2% (p=0.592), respectively. Table 3 presents the crosstabulation of eradication based on treatment.

**Table 3:** Crosstabulation of eradication based on treatment *p-value < 0.05 was considered statistically significant.

Treatment			Negative	Positive	p-value
Bismuth-Based Quadruple Therapy (n=409)	Gender	Male	113 (27.6%)	35 (8.6%)	0.482
		Female	191 (46.7%)	70 (17.1%)	
	Age	<50	171 (41.8%)	62 (15.2%)	0.619
		≥50	133 (32.5%)	43 (10.5%)	
Clarithromycin-Based Triple Therapy (n=783)	Gender	Male	223 (28.5%)	82 (10.5%)	0.012*
		Female	309 (39.5%)	169 (21.6%)	
	Age	<50	332 (42.4%)	167 (21.3%)	0.225
		≥50	200 (25.5%)	84 (10.7%)	
Levofloxacin-Based Triple Therapy (n=54)	Gender	Male	13 (24.1%)	6 (11.1%)	0.679
		Female	22 (40.7%)	13 (24.1%)	
	Age	<50	20 (37.0%)	11 (20.4%)	0.929
		≥50	15 (27.8%)	8 (14.8%)	

Odds ratios (OR) for the success of eradication were calculated for each treatment group. There were no significant indicators for the efficacy of BT on gender (OR: 1.18, 95% CI: (0.741, 1.889), p=0.482) or age (OR: 0.892, 95% CI: (0.569, 1.4), p=0.619). Age was not a significant indicator for the efficacy of CT (OR: 0.822, 95% CI: (0.598, 1.128), p=0.225); however, gender was a significant indicator (OR: 1.49, 95% CI: (1.093, 1.055), p=0.012). Significance indicates that gender plays a role in the success of this therapy. There were no significant indicators for the efficacy of LT based on gender (OR: 1.28, 95% CI: (0.39,4.22), p=0.679) or age (OR: 0.95, 95% CI: (0.30, 2.95), p=0.929) (Table 3).

## Discussion

Achieving effective eradication of *H. pylori* is paramount in the successful treatment of gastritis and peptic ulcers and the prevention of gastric cancer. However, antibiotic resistance poses a significant challenge, and some patients experience treatment intolerance. In contrast to other bacterial infections, determining antibiotic sensitivity for *H. pylori* treatment often necessitates empirical approaches due to limited local data on antibiotic resistance. This is attributed to the costs and time constraints associated with microbial culture, which is essential for conducting antimicrobial susceptibility testing. Therefore, it is essential to carefully select the most appropriate first-line eradication therapy for individual patients, considering the significant variation in sensitivity observed across different populations. Developing local reports on treatment efficacy is vital for guiding optimal treatment selection. Empirical therapy based on accurate medical history may be as effective as genotypic resistance-guided therapy [14]. Our study compared the effectiveness of empirical first-line treatment with BT for 10 days against CT and LT in *H. pylori* eradication. Although we observed a higher eradication rate with BT, the difference was not statistically significant.

Attaining a 100% cure rate for *H. pylori* infection remains an elusive goal in treatment planning. Currently, only a limited number of therapeutic regimens have consistently demonstrated eradication rates exceeding 90% [15]. According to Graham et al. (2013), a minimum of 90% eradication rate is generally considered the benchmark for effective *H. pylori* treatment [16]. However, achieving such high success rates is seldom observed in clinical settings, with treatment failures being relatively common. Our study revealed an overall eradication efficacy of 69.9% for our first-line eradication therapies, falling below the recommended 90% threshold. These findings, derived from real-world clinical practice, underscore the significant challenges posed by antibiotic resistance and noncompliance in *H. pylori* treatment.

Clarithromycin is a commonly used antibiotic in the treatment of various bacterial infections, including *H. pylori* infections. However, studies have indicated growing trends of clarithromycin resistance in the United States (US). The prevalence rates vary across different regions and populations. According to research conducted by the American Gastroenterological Association (AGA), clarithromycin resistance rates range from 15% to 40% in the US [17]. Several studies have demonstrated the effectiveness of CT in the US. A study conducted by Malfertheiner et al. in 2012 showed an eradication rate of 80% with 14-day CT [18]. Similarly, a meta-analysis by Liou et al. in 2016 reported a pooled eradication rate of 74.4% with CT [19]. On the other hand, Hassan et al. from Lebanon looked at the eradication rates of triple therapies, which showed suboptimal eradication rates (63.5%) when prescribing CT [20]. There are limited studies regarding the rise in clarithromycin resistance in Bahrain. However, a study conducted in Bahrain that examined 83 *H. pylori* isolates showed a 32.5% prevalence of clarithromycin resistance [11]. In our study, CT identified an eradication rate of 67.9%.

BT has gained substantial support as a first-line treatment option for *H. pylori* infection in the US, backed by many clinical studies and guidelines. This therapy has demonstrated high eradication rates, exceeding 85% and reaching up to 90% or more in some cases. Yang et al. reported 80-90% eradication rates with this regimen [21]. In our region, a study conducted in Saudi Arabia by Alsohaibani et al. demonstrated that BT exhibited a noteworthy efficacy in eradicating *H. pylori* infection. The study revealed a success rate of 78.3% in eradication, surpassing the efficacy of other treatment regimens utilized in the study [22].

Additionally, several studies have shown that the optimal regimen for *H. pylori* eradication should be decided regionally based on local antibiotic resistance and clinical practice data [23]. Another study by Afify et al. found that quadruple therapy was the best treatment with higher odds of eradication rate, and sequential therapy and quinolone-based therapy were associated with higher eradication rates compared to other regimens [24]. In Kuwait, Abd El-Naby et al., in a single-center study, have shown that BT is more efficient than CT (96% and 77%, respectively), while a study by Alboraie et al. showed 88% and 68.6% eradication rates, respectively, in a Kuwaiti population [25-27]. Our data have demonstrated that BT was superior to triple therapy, with an eradication rate of 74.3%.

According to the American College of Gastroenterology (ACG) guidelines, the use of levofloxacin therapy is considered an acceptable first-line option [17]. However, it is important to note that there has been an increase in resistance to levofloxacin, with rates reaching 31% in the US [27]. Several studies have evaluated the efficacy of LT in the US. These studies have reported eradication rates ranging from 75% to 90%, with variations depending on factors such as antibiotic resistance patterns and patient compliance [28]. A meta-analysis of seven trials conducted in different regions worldwide revealed comparable eradication rates between LT and CT (79% vs. 81%, respectively, risk ratio 0.97, 95% CI: 0.93-1.02) [29]. Another meta-analysis involving nine studies and 2502 patients supported these findings and highlighted regional differences in eradication rates, with LT being more effective in Europe and CT being more effective in Asia [30]. Data on levofloxacin resistance rates in Bahrain are limited. In our study, 35 out of 54 patients (64.8%) treated with LT eradicated *H. pylori* infection.

In October 2022, Alsohaibani et al. presented guidelines for managing *H. pylori* infection in Saudi Arabia. These guidelines were based on available data from prospective studies, which indicated that triple therapy and sequential therapy may not be the most effective treatments for *H. pylori*. Instead, the use of BT, such as Pylera®, was found to be superior as a first-line treatment [31]. However, in 2019, Alboraie et al. published Egyptian recommendations that favored the use of CT as the first-line treatment for *H. pylori* infection eradication in Egypt. The decision was based on studies that demonstrated good eradication rates ranging from 84.6% to 90% [32,33]. Levofloxacin, omeprazole, nitazoxanide (Alinia), and doxycycline (LOAD) achieved an 88% eradication rate when administered for 14 days and were recommended as a salvage therapy for patients who failed the first-line regimen [34]. Although BT had high eradication rates in both males and females (92% and 82%, respectively) in a 10-day regimen, it was not widely available in Egypt during the time of publication and, therefore, was only suggested if accessible [27,35].

Strengths and limitations

This study is the first to evaluate multiple *H. pylori* eradication regimens in the Kingdom of Bahrain, offering valuable region-specific data to inform clinical decision-making. The large sample size and use of validated diagnostic and follow-up tools (UBT and stool antigen tests) improve the internal validity of our findings. Data were retrieved from electronic health records, reducing recall bias and ensuring completeness for documented parameters.

However, several limitations must be acknowledged. First, the study was conducted in a single center in Bahrain, and the sample population may not be fully representative of other regions, both within and outside the country. Therefore, the generalizability of our results to broader or more diverse populations may be limited. Second, the retrospective design limits control over external variables, such as lifestyle factors, socioeconomic status, prior antibiotic exposure, or over-the-counter medication use, which may have confounded treatment outcomes. The study also did not include longitudinal follow-up beyond eradication, which may have allowed a better assessment of recurrence or delayed complications.

Additionally, there may be selection bias, as patients who did not return for follow-up testing were excluded. These individuals might differ in important ways (e.g., treatment adherence or side effects) from those who completed follow-up. Data collection bias was minimized using objective testing outcomes and standardized lab procedures; however, the reliance on clinician-documented compliance may still introduce some variability. Sampling was based on the consecutive inclusion of all eligible patients within the defined time frame (2017-2021), which improves internal consistency but may still limit external representativeness.

Although treatment compliance was assessed and factored into our analysis, we note that the extent and pattern of non-compliance varied widely. Some patients reported skipping a day or two mid-course, while others omitted doses sporadically or discontinued therapy near the end. Due to this heterogeneity and the lack of standardized documentation regarding the timing and quantity of missed doses, we were unable to subcategorize the non-compliant group in a meaningful way. Future studies should incorporate standardized tools for capturing medication adherence to allow more detailed stratification and outcome correlation.

Future studies would benefit from a prospective, multicenter design with control over confounding variables and longer follow-up periods to assess long-term outcomes.

## Conclusions

Our findings show that BT achieved the highest eradication rate at 74.3%, outperforming both CT (67.9%) and LT (64.8%). These results, drawn from a real-world outpatient population in Bahrain, reflect the growing challenge of antibiotic resistance and the declining efficacy of traditional triple therapies. Notably, even with the most effective regimen, the overall eradication rate in our study was only 69.9%, which falls short of the ≥90% threshold recommended for effective *H. pylori* treatment. This underscores an urgent need for local sensitivity testing and the development of national treatment guidelines tailored to regional resistance patterns. Additionally, our subgroup analysis demonstrated that non-compliance significantly reduced eradication success (34.7% vs. 76.2% in compliant patients, p < 0.0001), highlighting the importance of patient adherence and the potential utility of compliance-enhancing strategies.

While our retrospective design limited control over confounding variables, the study provides essential baseline data on eradication efficacy in the Kingdom of Bahrain. These results should prompt further prospective, multicenter research to evaluate alternative regimens such as sequential, concomitant, or LOAD therapies and to assess the feasibility and cost-effectiveness of routine microbial culture and antibiotic susceptibility testing. Ultimately, integrating these findings into clinical practice could improve eradication rates and help slow the spread of antibiotic resistance in the region.
